# A Simple Approach for Flexible and Stretchable Anti-icing Lubricant-Infused Tape

**DOI:** 10.1021/acsami.1c15634

**Published:** 2021-09-08

**Authors:** Marco Carlotti, Ilaria Cesini, Virgilio Mattoli

**Affiliations:** Center for Materials Interfaces, Italian Institute of Technology, Viale Rinaldo Piaggio 34, Pontedera 56025, Italy

**Keywords:** surface engineering, ice adhesion, anti-icing coatings, soot, lubricant-infused surfaces, slippery lubricant-infused porous surfaces

## Abstract

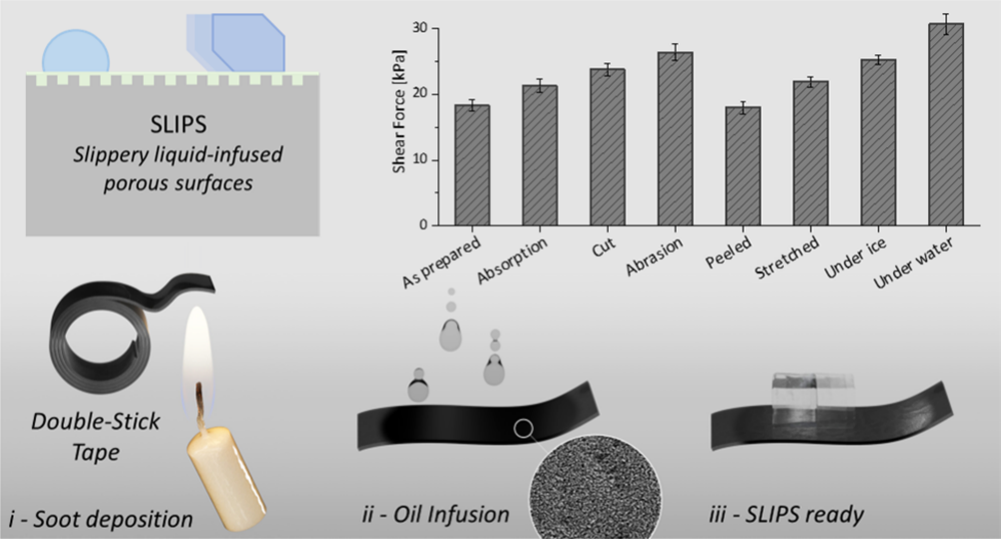

Unwanted icing has major safety and economic repercussions on human activities, affecting means of transportation, infrastructures, and consumer goods. Compared to the common deicing methods in use today, intrinsically icephobic surfaces can decrease ice accumulation and formation without any active intervention from humans or machines. However, such systems often require complex fabrication methods and can be costly, which limits their applicability. In this study, we report the preparation and characterization of several slippery lubricant-infused porous surfaces (SLIPSs) realized by impregnating with silicone oil a candle soot layer deposited on double-sided adhesive tape. Despite the use of common household items, these SLIPSs showed anti-icing performance comparable to other systems described in the literature (ice adhesion < 20 kPa) and a good resistance to mechanical and environmental damages in laboratory conditions. The use of a flexible and functional substrate as tape allowed these devices to be stretchable without suffering significant degradation and highlights how these systems can be easily prepared and applied anywhere needed. In addition, the possibility of deforming the substrate can “allow” the application of SLIPS technology in mechanical ice removal methodologies, drastically incrementing their performance.

## 1 Introduction

Anti-icing surface technologies, which oppose ice formation and ease its detachment, could have a huge impact on the global economy, affecting disparate aspects of our life such as transportation (aerial, marine, and land-based), energy production and distribution, infrastructure maintenance and damage mitigation, and consumer goods.^1,2^ The presence of unwanted ice on surfaces increases weight-related stress and air friction, resulting in wear and increased energy demands. In the case of operated carriers, it also lowers maneuverability, thus increasing the risk of accidents. The most common commercial deicing solutions consist of infrared or electrothermal melting,^3^ addition of low-freezing point agents,^4^ pneumatic actuation,^5^ and mechanical vibration,^6^ which require a high energy demand and need to be engineered for specific applications rather than offering a broadband solution.

**Figure 1:**
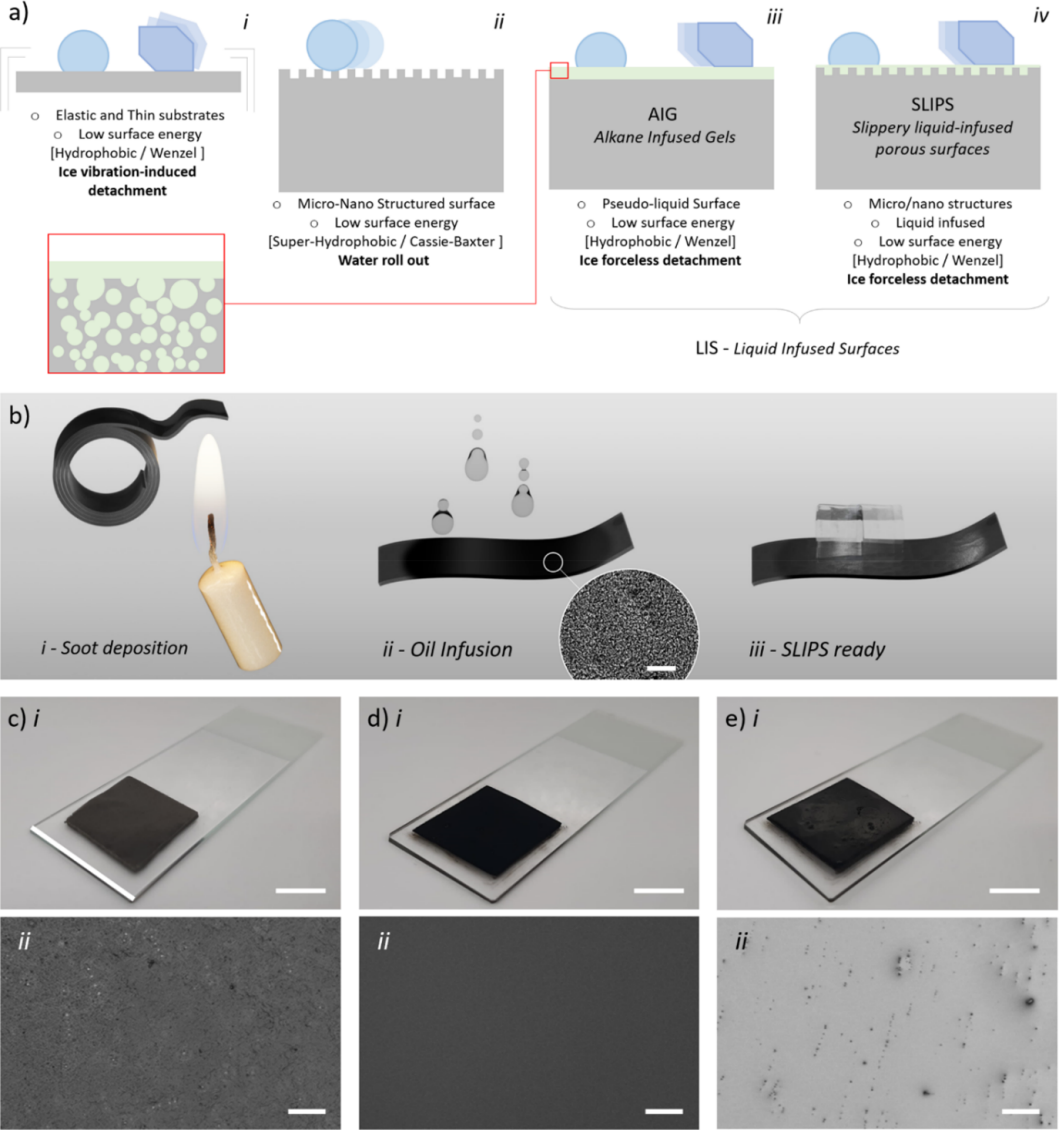
(a) Schematics of inherent anti-icing surfaces and brief descriptions of working principles. (b) Fabrication method for stretchable SLIPS comprising candle soot and liquid lubricant on double-sided tape as a substrate (inset SEM image scale bar, 20 μm). (c–e) photographs (i) and optical profilometry (ii) of, respectively, the tape substrate (3M), as-deposited soot, and as-prepared SLIPS after addition of silicone oil. The scale bars represent 1 cm and 100 μm in the camera picture and the optical profilometry image, respectively.

In the past decades, however, research has instead focused on a different kind of anti-icing technology by devising intrinsically icephobic surfaces (Figure 1a).^7,8^ These “passive” methods solely rely on the physical and chemical properties of the surface to obtain low affinity to the forming ice, and thus, they can be highly cost-effective, environmentally friendly, and horizontally translated to many different fields of application.^9^ Many different approaches were proposed during the years, each with their advantages and drawbacks.^9^ For example, hydrophobic materials and coatings (including molecular monolayers), by lowering the wettability of water, can promote passive ice slushing, although often with limited performance.^10−12^

Nanostructured superhydrophobic surfaces (SHSs) can prevent ice formation by limiting the water contact time. Such properties are purely morphological and thus can be obtained by etching, controlled growth, or lithographic approaches on a broad range of materials regardless of their chemical nature.^13−19^ In addition, one can treat these surfaces in subsequent steps to increase their performance further (e.g., modification with a molecular monolayer).^20^ However, such rough surfaces are fragile and prone to damage, and they favor heterogeneous ice nucleation and increase the effective ice adhesion area once ice forms.^21^

An alternative solution could arise from liquid-infused surfaces (LISs).^22,23^ These consist of surfaces that are covered with a thin layer of lubricant, which lowers the surface energy and makes it ultraflat. In this way, liquids can slip away at very low tilt angles (usually <5°) and ice can detach with extremely low forces.^24^ This observation is in agreement with the equation
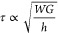
1which describes the ice shear adhesion force (τ) as proportional to a function of the work of adhesion (*W*), the shear modulus of the substrate (*G*), and its thickness (*h*).^7^ In the case of LISs, with the substrate being a liquid, *G* may be considered infinitesimally small, drastically reducing τ even for thin lubricant layers.

Depending on where the lubricant reservoir is located, they can be divided in two categories: alkane-infused gels (AIGs) and slippery liquid-infused porous surfaces (SLIPSs).

The former consists of soft polymer networks infused with long chain alkanes throughout their volume.^25−29^ In these systems, the alkane wax, which could also be substituted with other compounds, such as (per)fluorinated liquids,^29^ to enhance the properties of the material, will tend to migrate toward the interface and form a thin icephobic layer that can easily replenish itself when needed (in response to damage or lubricant leaching).^30^ AIGs are therefore very robust systems also capable of achieving low ice adhesion forces. In addition, smart nanotechnological solutions can improve the performance of these materials and the control on their properties even further.^31−33^

Conversely, SLIPSs consist of highly porous nanostructured materials where the lubricant fills the cavities and covers the surface, resulting in an interface very similar to that of AIGs.^34^ They are commonly prepared from micro/nanostructured SHS by impregnation with suitable high-boiling liquids that replace the air. The capillary forces hold the lubricant in position, minimizing the flow and unwanted release. Moreover, the slippery properties can combine with other surface behaviors like photothermal generation to increase the overall efficiency of the system.^8,35^

One advantage of SLIPSs over AIGs is that the former can be obtained from a broad variety of nanostructured materials, such as polymers, carbon, metal oxides, metals, and even paper, and thus, they appear as a very versatile solution to achieve icephobicity in diverse applications.^36−40^ This versatility also translates in the possibility of preparing extremely low-cost designs, ideal for low-end applications.

A viable option in this sense is provided by candle soot, which can be obtained cheaply and reliably anywhere.^41^ When deposited directly from the flame, it forms a complex nanostructured material characterized by a well-defined fractal structure that renders the surface superomniphobic (i.e., it has extremely high contact angles for both water and organics and low droplet contact time).^42,43^ Several groups already explored the anti-icing properties of candle soot as an SHS,^19,44,45^ as a photothermal deicing platform,^41,46^ and as a host for fabrication of SLIPS,^47^ highlighting the stability of such systems, their simple fabrication procedures, and their tolerance to damage. SLIPSs in particular are tolerant to several forms of damage because of their fractal structure, the mobility of the lubricant that can quickly fill vacancies, and the interfacial forces between the lubricant and the soot that makes the host matrix more tolerant to compression damage.^47,48^ Such characteristics suggest that by using a proper substrate, SLIPS surfaces could deform and stretch to a certain degree without suffering loss of performance, an incredible added value for the realization of rapid and on-demand anti-ice fixings. However, to the best of our knowledge, there are no examples of flexible SLIPSs in the literature, nor their possible advantages have been explored.

In this study, we show how it is possible to realize a candle soot-based SLIPS using stretchable double-sided tape as a support and silicone lubricant, thus resulting in a deformable icephobic coating that can be applied on any structure on demand without altering its properties. Because of its simplicity, anyone can prepare such a system easily and rapidly using common household items, thus making this approach incredibly useful for practical low-end applications. In addition, we investigate the effect of the addition of a fluorinated molecular monolayer and the use of a halogenated lubricant on the overall anti-ice properties and their stability under diverse types of stress. Finally, we show that the flexible nature of these SLIPSs, which in addition offer an inherent low adhesion force, can produce the detachment of the ice employing extremely small strains and applied forces, underlining the potential of this technology even for higher-end applications.

## 2 Experimental Section

### 2.1 Materials

3M double-sided tape (3M, VHB 4611, acrylic foam-based) and polyurethane-based double-sided tape (PU, LIUMY Multipurpose NanoGrip) were acquired on Amazon.it. Candles were obtained from a local hardware store in Pisa (Italy). Silicone oil (viscosity, 10 cSt; surface tension, 20 mN m^–1^) and 1*H*,1*H*,2*H*,2*H*-perfluorooctyltriethoxysilane were purchased from Merck. Halocarbon 6.3 oil (viscosity, 6.3 cSt; surface tension, 23 mN m^–1^) was kindly provided by Solvadis Specialties GmbH (Germany).

### 2.2 Preparation and Characterization of the SLIPSs

A piece of double- sided tape was attached to a support and exposed to the top part of a candle flame for a total of 60 s divided in 10 s intervals. After allowing the samples to cool to room temperature, unattached soot was removed by a gentle blow of air, and a few drops of the chosen lubricant were added. The drops rapidly flattened on the surface and covered it entirely. The SLIPSs were then left in a vertical position so that the excess lubricant collects on the bottom edge and can be easily removed with paper towel.

In the case of soot functionalized with the fluoroalkyl-silane, after the soot deposition step, the samples were placed in a desiccator at 200 mbar with a vial containing a few drops of the silane for 1 h. The rest of the fabrication procedure was identical to what was reported in the paragraph above.

Optical images of deposited soot and SLIPS samples were acquired with a Hirox Digital Optical Microscope KH-7700. Scanning electron microscopy images were obtained with a FIB/SEM Helios Nano-Lab 600i (FEI), with 5 kV electronic beam acceleration at different magnifications. Optical profilometry was performed by a Leica DCM 3D Confocal Profilometer at 10× and 150× magnifications. The analysis of the optical profilometry images was performed using Gwyddion (v2.57). Water contact angle measurements were performed by means of an optical tensiometer model Theta (Biolin Scientific). The droplets’ moving speed was measured by analyzing video recordings (Adobe Premiere 2017) of droplets sliding on 5.5 cm-long flat SLIPSs with different inclinations.

### 2.3 Evaluation of Icephobic Properties

A plastic Eppendorf vial with a diameter of 1 cm and a height of 3 cm was filled with water to the brim and placed upside down on the samples. These were moved to a freezer at −21 °C, where the samples were left for at least 3 h. Shear force measurements were performed in the freezer directly or immediately after removal from the freezer using a dynamometer (DGD-7, Mecmesin, UK).

### 2.4 Stability and Damage Tests

For each test, three samples were individually prepared (following the procedure in Section 2.2). After the damage was performed, all the samples were measured in the same conditions reported in Section 2.3 for four cycles. One sample was tested 2 months after preparation.

#### 2.4.1 Water Immersion

As-prepared SLIPSs were placed in a plastic container filled with tap water for a period of 2 weeks. Every 3 days, the water was changed.

#### 2.4.2 Immersion in Ice

As-prepared SLIPSs were placed in a plastic container filled with deionized water for a period of 2 weeks. The ice was then melted at room temperature, and the samples were extracted.

#### 2.4.3 Cuts

As-prepared square-shaped SLIPS samples were cut with a kitchen knife to make two perpendicular lines crossing at the center. The kitchen knife was chosen as it was less sharp than a razorblade or a scalpel.

#### 2.4.4 Lubricant Absorption

As-prepared SLIPSs were covered with absorbing paper (Coop Spa, Italy, 100% cellulose) and left for 10 min with a 40 g weight over them.

#### 2.4.5 Abrasion

As-prepared SLIPSs were covered with sand paper (average grain, 320; acquired from a local shop in Italy) with a 40 g weight over them. The sand paper was then slit away with the weight still on.

#### 2.4.6 Peeling with Tape

As-prepared SLIPSs were covered with tape and left for 2 min with a 40 g weight over them. The tape was then peeled.

#### 2.4.7 Pressure

Employing a steel cylinder, 100 kPa (over a surface of 1 cm^2^) was applied on as-prepared SLIPSs for 1 min. The pressure was then released, and the sample was recollected.

#### 2.4.8 Stretching

SLIPSs were prepared on 3M double-sided tape samples attached to two different supports, which enabled the stretching. After the usual preparation procedure, the surfaces were stretched three times of about 30% (higher than the elastic strain of 3M, which we measured to be about 5%) before being transferred to a rigid substrate to facilitate the measurement.

### 2.5 Finite Element Simulations

A 3D solid mechanics model was built in Comsol Multiphysics v5.6 to analyze the force/strain necessary to detach the ice from the 3M tape. The geometry and material properties of the 3M tape and the ice column are reported in the Supporting Information (Figure S10a). The Young modulus of the 3M tape was set to 450 kPa, as defined by the supplier, while the ice properties were taken from the software materials library. Both the 3M tape and the ice were modeled as homogeneous, linearly elastic materials. The right-end face of the tape was defined as a fixed constraint boundary, while the structure was pulled at a constant pressure, *Ps*, from the left side, as shown in Figure S10b. The displacement of the tape along the *z* axis was prevented by a roller constraint applied to the bottom surface of the structure. A free tetrahedral normal mesh was generated, and the number and size of the elements were refined at the ice–tape interface. A stationary analysis was performed using the default solver settings, and a parametric sweep was implemented to investigate the effect of varying *Ps* in the range of 10–100 kPa, with a step of 10 kPa. A similar study was carried out with pressure applied at the bottom of the ice column, as reported in the Supporting Information.

## 3 Results and Discussion

### 3.1 SLIPS Design and Fabrication

The fabrication of SLIPSs to work as icephobic media requires an accurate design of the system to provide reliable working performance. All the different interactions involved—surface–lubricant, lubricant–water, and surface–water—must be balanced to weaken the ice adhesion while, at the same time, limiting the damage to the surface and lubricant leakage.^49−51^ To do so, the porous surface that serves as a host must be mechanically robust, have a large specific surface, and be affine to the chosen lubricant to be easily impregnated while efficiently repelling water. Despite the simple preparation method, candle soot revealed to be an ideal substrate for the fabrication of surfaces with such characteristics.^47^ In Figure 1b, we show a schematic of the method used to prepare a SLIPS using candle soot; images of a SLIPS sample at different preparation steps are also shown in Figure 1c,d.

Soot interaction with the substrates is generally weak and necessitates a binding agent. In our study, we decided to use adhesive tape as it is a soft and stretchable substrate, which, at the same time, provides the necessary adhesive properties. Other reports of soot-based SHSs and SLIPSs in the literature make use of a binding layer that is spin-coated on the substrate prior to the deposition, a fabrication step that prevents the application of these technologies to nonflat surfaces and incompatible materials.^46,47^ Moreover, one can conveniently find it in almost every household and, once treated, can be applied directly where necessary, making it an approachable solution to cheap and readily available icephobic coatings for common applications.

**Figure 2:**
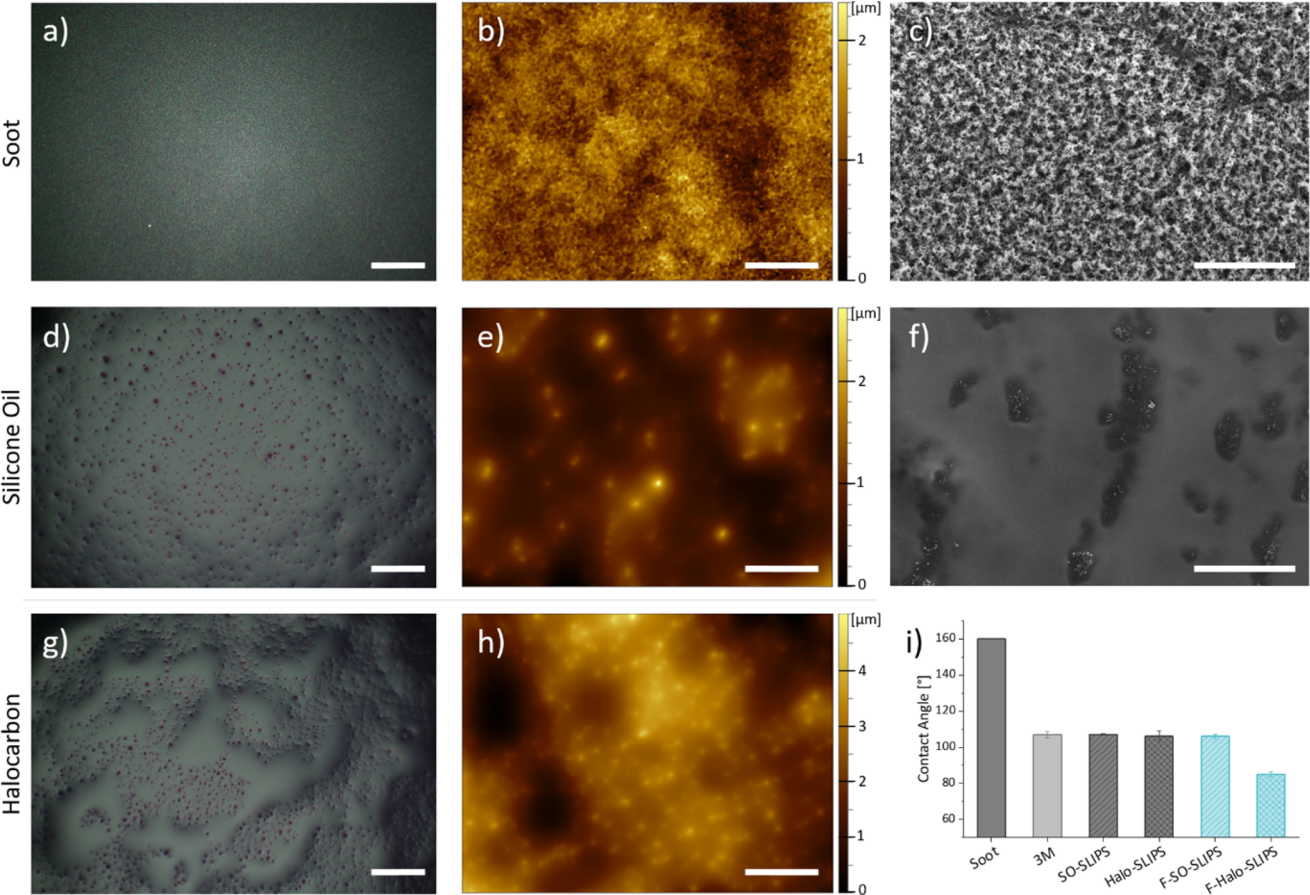
Images of candle soot deposited on 3M tape: (a) optical microscopy, (b) optical profilometry height map, and (c) SEM. Images of silicone oil-impregnated SLIPS: (d) optical microscopy, (e) optical profilometry height map, and (f) SEM. Images of halocarbon-impregnated SLIPS: (g) optical microscopy and (h) optical profilometry height map. (i) Static water contact angle for different substrates. Scale bars represent 100, 50, and 10 μm for optical microscopy images, profilometry height maps, and SEM pictures, respectively.

By exposing the tape to the top part of the flame of a candle for a brief period of time (about 1 min), we obtained a black coating of soot nanoparticles (diameter below 50 nm), which is about 12 μm thick (Figure S1), characterized by a well-defined fractal structure of channels down to nanometric resolution. We report an example of these surfaces in Figure 2a–c, showing that it is similar to soot depositions in another study.^46^ The Minkowski–Bouligand fractal dimension associated with these surfaces was calculated to be between 2.7 and 2.9 (from the 1200 μm^2^ and 0.03 mm^2^ SEM pictures in Figure 2c and Figure S2, respectively), indicating its complex evolution in space.^43,52^ Such morphology is responsible for the observed superhydrophobicity of soot-covered substrates, which can trap air when immersed under water, resulting in the mirror effect typical of the Cassie–Baxter regime (Figure S3).^48^ However, in the case of SLIPSs, it also aids in the dispersion and the retention of the lubricant by means of increased capillary forces and a larger specific surface. In addition, the small channels limit the flow of the lubricant, preventing dripping and reducing the pressure damage (Figure S4).

Next to the host matrix, the choice of the liquid phase is also of paramount importance as it should not be readily miscible with water and able to wet the porous surface.^50^ In our investigation, we decided to evaluate both a silicone oil (SO-SLIPS) and a commercially available fluorinated lubricant (Halo-SLIP) of similar viscosities in the fabrication of icephobic SLIPSs. Silicone oil is commonly used in SLIPSs and AIGs as it readily forms slippery flat surfaces and is immiscible with many liquids, while fluorinated compounds are known for their low surface energies and affinity toward both organics and water.^29^

In the case of both liquids, it was possible to impregnate the soot layer, obtaining smooth surfaces as shown in Figure 2. We characterized the latter using optical microscopy, SEM, and optical profilometry. We found the surfaces to be relatively flat for both SO- and Halo-SLIPS, with large liquid domains and a few protruding tips of the underlying soot network, which are about 1–3 μm tall. Unfortunately, it was not possible to obtain SEM photos of the Halo-SLIPS as the boiling point of the fluorinated lubricant was too low to sustain the high vacuum. While the two surfaces look very similar, the one fabricated with silicone oil seemed to have less defects and lower roughness (mean square roughness on 0.05 mm^2^ area: 202 nm vs 437 nm of the Halo-SLIPS), pointing to the fact that the soot matrix interacts better with the nonhalogenated lubricant. This low roughness also correlates with the lower fractal dimension (of about 2.1) calculated for SLIPSs when compared to as-deposited soot, indicating an almost flat surface (see Figure S5).

We measured the water contact angles (θ) to be of about 106 ± 1° for all the SLIPS samples (with the exception of the F-Halo-SLIPS), similar to that of bare 3M and much lower than that of as-deposited soot (Figure 2i). A minimal inclination (<2°) was enough to provoke the slippage of the water as we show in Figure S3. This observation also points to the fact that the advancing and receding contact angles are similar to their static value.^53^ One may use the values of θ to make considerations about the ice formation mechanism in the samples. For example, following the classical nucleation theory, one may describe the relation between the free-energy barrier for heterogeneous and homogeneous nucleation (ΔG*_heter_ and ΔG*_homo_, respectively) using the equation
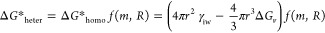
2where *r* is the nucleation germ radius, γ_iw_ is the surface energy at the water–ice interface, Δ*G_v_* is the volumetric free-energy variation that accompanies the ice formation, and *f*(*m*,*R*) is a function that represents a geometrical correlation factor that takes into account the effect of external bodies in facilitating the nucleation (i.e., lowering the nucleation barrier) at the water interface.^54,55^ The parameter *m* in the latter is related to the interfacial energies between the liquid water, ice, and the substrate, and it is proportional to cos(θ). *R* is a parameter that depends on the ratio between the size of the heterogeneous nucleation sites and the critical nucleation radius: for a flat substrate, as it is the case of SLIPS, *R* tends toward infinity and *f*(*m*,*R*) can be considered a function of only θ
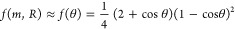
3

As θ increases toward 180°, *f*(*m*,*R*) approaches unity (homogeneous nucleation limit), and the homogeneous and heterogeneous nucleation paths become energetically equally probable. In the specific case presented here, *f*(*m*,*R*) ≈ 0.7 for the SO-SLIPS on 3M, thus showing that homogeneous nucleation is not energetically prohibited.^47^ This value for the SLIPS is taken from experimental values of θ and, as such, includes the presence of intrinsic defects on the surface (as seen in Figure 2). To clarify, it is worth mentioning that the large θ value measured for the soot as deposited, which is characterized by superhydrophobic properties (θ > 160°), is due to the Cassie–Baxter regime and cannot be used in the previous equation as it is related to the morphology of the substrate rather than the expression of the interfacial forces between the different phases. For superhydrophobic surfaces, heterogeneous nucleation is usually favored, which increases the ice adhesion strength to the surface.^7,9,34^ However, while the classical nucleation theory can be useful to rationalize the dynamics of ice formation on SLIPS, its thermodynamic and kinetic details are far from being precise, and as such, the conclusions presented herein should not bear any quantitative value.^56^

To provide an environment more compatible with the halogenated oil, we tried to functionalize the soot layer with a monolayer of a fluorinated alkylsilane immediately after deposition (F-SO-SLIPS and F-Halo-SLIPS).^57^ In this way, the surface chemistry inside the pores changes drastically while still maintaining its original morphology. Optimizing the wetting between the lubricant and the matrix is of paramount importance since, otherwise, a liquid with high surface energy as water will tend to displace the former, affecting the durability of the system. We discuss the importance of the balance between the different interface energies—water–lubricant, matrix–lubricant, and water–matrix—in the next section.

As anticipated, with the functionalization, the F-Halo-SLIPS resulted in a lower local roughness (342 nm). Conversely, the F-SO-SLIPS presented an increased amount of corrugation and an increased number of defects (Figure S6), which are evidence of the worse wetting between the fluorinated host matrix and silicone oil (roughness, 360 nm). The SEM analysis, however, showed that the morphology at the micrometer scale is not much different than that of the SO-SLIPS. The passivation with the silane also seemed to reduce the water contact angle in the case when halocarbon is used as the lubricant (Figure 2i), indicating that heterogeneous nucleation may occur more easily on this type of substrate (ΔG*_heter_ ≈ 0.4 ΔG*_homo_).

### 3.2 Evaluation of Icephobic Performance

As introduced previously, SLIPS and AIG interfaces are covered with a thin layer of lubricant that increases the smoothness and prevents ice adhesion by reducing nucleation sites. This affects both frost formation and the shear force necessary to remove the ice.

**Figure 3:**
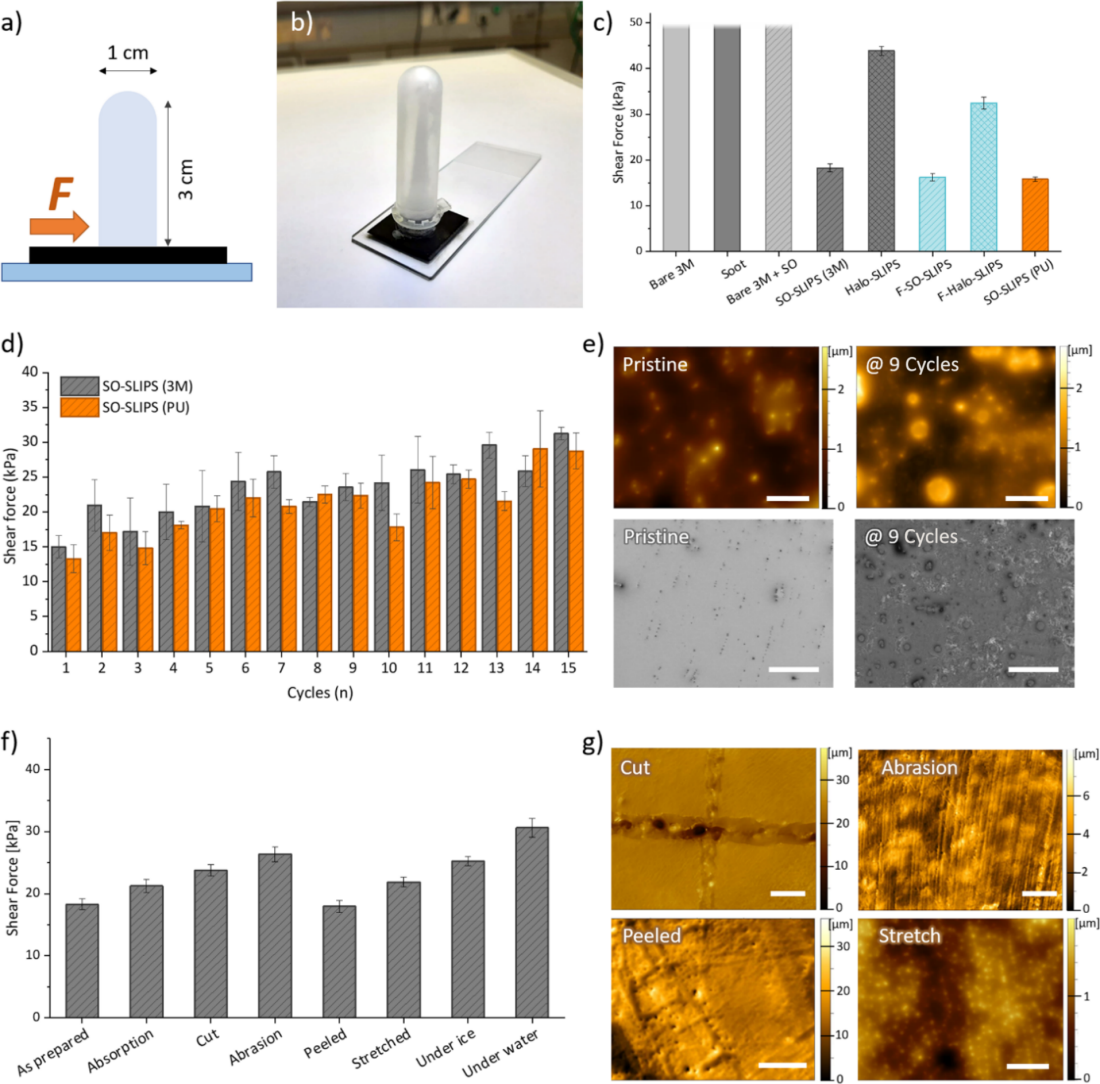
(a) Schematic representation of the shear force measurement. (b) Example of an ice column on the SLIPS used in the experiments. (c) Summary of ice shear adhesion forces measured on different substrates (the left most columns are out of range, >50 kPa). (d) Plot of ice shear adhesion strength over multiple cycles for SLIPS prepared using 3M and PU as substrates. (e) Comparison of optical profilometry images and height maps for pristine SO-SLIPS and after nine icing-removal cycles (scale bars represent 50 and 100 μm for profilometry height maps and optical microscopy images). (f) Ice shear adhesion forces measured for differently damaged SO-SLIPS. (g) Optical profilometry height maps for these latter surfaces. Scale bars in this figure represent 200 and 50 μm for the “Stretch” example in panel (g).

We evaluated the ice adhesion performance of all the surfaces discussed so far by measuring the shear force necessary to remove a column of ice with a diameter of 1 cm.^7^ Such a method is often used in the literature to compare the performance of icephobic surfaces. The results we obtained are summarized in Figure 3. As we show, SLIPSs comprising soot and silicone oil were characterized by the lowest ice adhesion forces below 20 kPa. This threshold is usually mentioned in the literature as the minimum requirement to achieve ice detachment under environmental conditions without human intervention.^1,7,10,30^ Remarkably, the SLIPS surfaces investigated in this study met this requirement despite being fabricated from everyday household items such as double-sided tape, a candle, and lubricating oil. Notably, most times when removing the ice column, we observed minimal soot transfer, indicating the stability under these conditions (Figure S7). The silanization of the soot or change of the substrate to a different kind of double-sided tape (polyurethane-based, PU) did not affect significantly the icephobic properties of the SO-SLIPS system.

The use of the fluorinated lubricant (Halo-SLIPS series) resulted in less performing surfaces, a reflection of the high number of defects reported in the previous section, which offers a higher chance of heterogeneous nucleation. Different from the SO case, for the halocarbon–soot system, the modification with the fluorinated alkylsilane sensibly reduces the ice adhesion strength. However, such improvement is not sufficient to meet the 20 kPa target. The lower performance of halocarbon as the lubricant may be ascribed to the lower surface energy associated with fluorinated materials. While the latter is responsible for the inertness that makes such materials appealing, it also reduces the extent of interaction with the host matrix, allowing an easier displacement of the lubricant by the water. This can be rationalized with the spreading parameter, *S*_ab_, which defines the tendency of phase a to penetrate phase b, and it is expressed by the relation
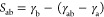
4where γ_a_ and γ_b_ represent the surface energies of phases a and b, respectively, and γ_ab_ is the interfacial energy between them.^58^*S*_ab_ < 0 indicates that the two phases do not penetrate each other (as that would result in an increment of the total energy by means of a more extended high-energy interface).^48,50,59^ Following the approach of Preston et al.,^50^ we could estimate the values of *S* for the different water–lubricant couples as
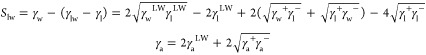
5where the subscripts l and w indicate the lubricant and water phase, respectively, while the LW, *+*, and – superscripts represent the dispersion forces and polar (considered as Lewis acid–base interactions) contributions, respectively. For silicone oil–water, γ_l_^LW^ and γ_w_^LW^ of 20.1 and 21.8 mN/m,^50^ respectively, for which one can safely assume no polar contributions,^50^ we found *S*_lw_ to be about +1.6 mN/m, indicating that a slight cloaking of the water droplets by the lubricant may happen.^23^ This could also be the reason why the tape–soot SLIPSs presented herein have slightly worse performance than other similar soot-based ones (which have been shown to reach adhesion forces of about 2 kPa).^47^ Conversely, when halocarbon oil is considered, despite the higher γ_l_ (23 mN/m), the polar interactions between the lubricant and water (described by the third term in eq 5) make the penetration and displacement relatively more likely in this case.^50^ The anti-icing performance of tape–soot SLIPSs could benefit from the use of more viscous, apolar lubricants;^23^ however, such optimization was out of the scope of this paper.

### 3.3 Stability Tests

For their planned application, anti-icing surfaces are subjected to strong environmental conditions, which may compromise their function. To address this issue, we performed several stability tests of our SLIPS, monitoring the change in ice adhesion strength, and the results are summarized in Figure 3f,g.

Retention of the lubricant is a major issue as, in the case of most SLIPSs, surface interactions are the only forces that prevent leaking. Candle soot revealed to be a well-performing substrate for silicone oils, and even absorption tests performed using adsorbent paper resulted in a relatively small loss in performance, which could as well be attributed to mechanical damage.

Indeed, direct mechanical solicitations can damage the host structure, affecting the retention of the lubricant and smoothness of the surface. However, the mobile nature of the liquid phase and its affinity to the soot matrix, together with the fractal structure of the latter, can adapt remarkably well to minor damages^48^ and even show self-healing properties.^57,60^ Mostly, they result from the tendency of the lubricant to migrate from the reservoir to the surface to minimize its surface tension.^27^ We performed cut and abrasion tests as described in Section 2. As expected, these types of damages affected the ice adhesion of the SLIPS, which, however, remained below 30 kPa, much lower than the control experiments on bare 3M tape, soot, and 3M tape treated with silicone oil. These results show that the lubricant can efficiently flow around the reservoir and replenish the damaged surface, as supported by the optical profilometry images in Figure 3g and Figure S8. While this mechanism can repair small damages, it does not prevent the degradation of the soot matrix. One can directly observe this from the lower and nonconstant sliding velocity of water droplets on the damaged surfaces (Figure S3), indicating a nonuniform surface.

Application of high pressure (100 kPa) was found to not affect significantly the ice adhesion characteristics of the surfaces (20.2 ± 0.4 kPa). The presence of the lubricant in the soot matrix (rather than air) effectively makes it more tough and resilient, as it usually happens in composite materials, and thus better able to withstand pressure damage. Indeed, images comparing the deposited soot and SLIPS surfaces after the application of a vertical force show the differences in the mechanical strength of these layers (Figure S4). In particular, such pressure was sufficient to compress and remove the soot layer alone; however, in the case of a SLIPS, the damage was found to be much more contained.

We also investigated the effects of adhesive tape on the SLIPS surface since such a test is able to remove soot efficiently from different surfaces (Figure S7). Profilometry images show that after the removal of tape, the surface appeared slightly smoother (mean square roughness of 189 nm vs 202 nm of the SO-SLIPS as prepared), as if the adhesive tape could efficiently remove the soot defects protruding from the lubricant. The anti-ice performance of the SO-SLIPS was not affected by the peeling (even after multiple cycles). Thus, in principle, one may use adhesive tape to protect and store the tape–soot SLIPS between preparation and application.

Because of the interface dynamics discussed in the previous section, we studied the stability of the SO-SLIPS under water and ice for prolonged periods of time. After being left for 2 weeks under water or solid ice, the performance of the tape–soot SLIPSs resembled that of the aforementioned mechanically damaged ones. In particular, treatment with water for prolonged periods seemed to affect the SLIPS properties the most. We ascribed this evidence to the low value of *S*_lw_ for the SO-SLIPS previously discussed. However, even in this case, we measured the ice adhesion to be about 30 kPa, a good value for practical low-end application and an optimal starting point for further optimization. In comparison, aging in air for a period of 2 months did not affect significantly the ice adhesion strength (21.7 ± 0.57 kPa).

Consecutive icing–deicing cycles may also affect the long-term stability by slowly removing the lubricant and damaging the soot structure. We therefore exposed the SO-SLIPS to sequential freezing cycles, continuously monitoring their performance. Notably, after 15 cycles, the ice adhesion strength was still below 30 kPa, a value similar to those measured for the other types of damages but that still allows ice detachment with minimal effort. A closer look on the surface showed that the protruding defects did not increase in height but rather became larger, as if they were slowly merging together over cycles (Figure 3e).

This performance is comparable to what has been observed in similar systems tested in laboratory conditions.^28,39,47^ However, in real applications, the properties and the stability of SLIPSs (including the ones presented herein) could differ as the mechanical stability of the soot matrix is limited when compared to other anti-icing technologies (see Table S1).

Nonetheless, it is worth mentioning that in the case of damage that compromises the icephobic function of the coating, the cheap and rapid fabrication procedure allows for its swift substitution. Such an approach is not possible (or, at least, it requires much more effort) in the case of SLIPSs or other icephobic surfaces with more complex fabrication methodologies, despite the fact that it might show better performance during the tests.

### 3.4 Stretchability

One distinctive feature of the SLIPSs that use tape as a substrate is their intrinsic stretchability. Both the tape and the fractal micro/nanostructure of the soot can easily sustain stretching and bending, and the addition of a lubricant is not expected to change this behavior. Remarkably, stretch cycles up to 30% (well beyond the elastic limit of the 3M tape, which is about 5%; Figure S9) did not affect the ice adhesion characteristics of the SO-SLIPS significantly. Optical profilometry data and SEM imaging support this finding, underlining the limited effects of stretching on the surface morphology (Figure S9). This feature allows anyone to prepare the tape–soot SLIPS easily and apply on demand when and where deemed necessary, which is no different than the application of regular double-sided tape.

**Figure 4:**
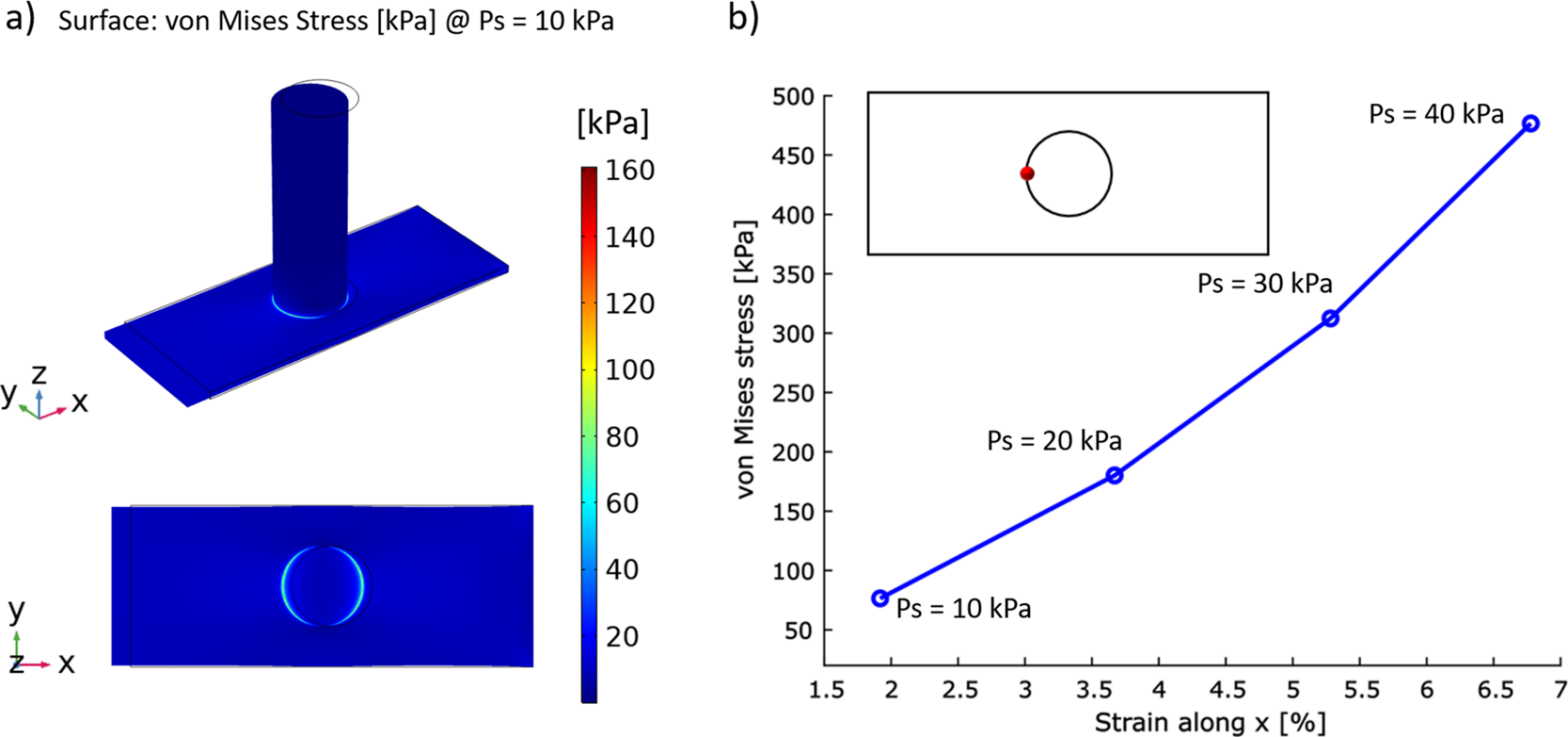
Results of the finite element simulations. (a) Von Mises stress distribution in the 3D geometry (top) and on the *xy* top plane of the ice–tape system (bottom) when the tape is pulled at 10 kPa. (b) Evolution of the von Mises stress at the ice–tape interface (red dot) as a function of the applied strain in the range of 10–40 kPa.

In addition, the use of stretchable and elastic substrates can allow the removal of ice by means of deformation of the substrate itself rather than applying a force on the ice formations. Such methods are employed, for example, on small aircraft to efficiently detach ice from the wings.^5^ The application of SLIPS technology, which passively offers a low ice adhesion, to such devices could improve their effectiveness and limit their energy consumption. In trying to investigate this possible application, we performed some simulations to check the stretching necessary to detach ice from SLIPS realized on 3M tape. Figure 4a shows the von Mises stress distribution in a 3D view and on the *xy* plane of the ice–tape system, obtained at 10 kPa (the evolution of the von Mises stress as a function of the full range of pressure values is reported in Figure S10). The stress was maximized at the interface between the two materials in the direction of application of the load. As reported in Figure 4b, the minimum applied load of 10 kPa, corresponding to a 2% strain of the tape extremity (extracted from the longitudinal displacement displayed in Figure S10), resulted in a von Mises stress of 77 kPa at the ice–tape interface (measured at the red point in Figure 4b).

These observations point to the fact that in the case of longitudinal stretch, a very small strain (less than 1%) is already enough to generate a force sufficient to detach the ice, making stretching a viable solution for ice detachment. Besides the longitudinal strain, we also investigated other types of deformations, finding that they were equally efficient if not better performing as in the case of a force applied from below the surface (see Figure S11). We tried to validate these findings experimentally; however, the strain values were too small to be measured as the ice columns would detach as we started the drawing of the SLIPS. This observation, however, supports the results of the numerical calculations and shows the potential of stretchable SLIPS to make practical ice removal technologies compared to rigid surfaces.

In synthesis, despite the use of common household items, the proposed SLIPSs showed anti-icing performance comparable to other systems described in the literature, a good resistance to mechanical and environmental damages, and the possibility to exploit additional properties such as adhesiveness/conformability and stretchability. A qualitative comparison of the proposed solution with respect to the most relevant anti-ice technologies at the state of the art is also briefly summarized in Table S1 for reference.

## 4 Conclusions

In this study, we fabricated and characterized the anti-icing properties of SLIPSs comprising everyday household items such as double-sided tape, candle soot, and silicone lubricant. As such, these systems are intrinsically of low cost, easy to fabricate, readily available, and scalable, which makes them a good option for low-end, on-demand applications with a broad scope. Remarkably, despite the simple fabrication procedure, the performance of these systems was comparable with that of other SLIPSs found in the literature, resulting in ice adhesion forces below 20 kPa for SLIPSs obtained by impregnating the soot–tape substrate with silicone oil. In laboratory tests, we observed that exposing such systems to several damages and harsh conditions—cuts, abrasion, peeling, prolonged immersion in ice or water, and freezing cycles—resulted only in a limited loss of performance. In particular, the soot–tape SLIPS, being prepared on elastic substrates, could undergo stretching cycles without degradation and, unlike most examples found in the literature, make possible the efficient exploitation of stretching and bending motions to detach the ice (effective even at very small strains, i.e., in the order of 1%).

Finally, we believe that the SLIPSs here presented offer a simple and effective solution for an anti-icing system that can be applied when and where needed with the simplicity of applying scotch tape while, at the same time, offering good performance.

## Supporting Information Available

Figure S1: optical profilometry analysis of candle soot deposited on double-sided tape; Figure S2: SEM picture of deposited soot and fractal dimension extraction; Figure S3: SEM images of deposited soot and SO-SLIP and hydrophobicity confederations; Figure S4: optical microscopy images of sample subjected to pressure damage; Figure S5: optical profilometry map for SO-SLIPS and fractal dimension extraction; Figure S6: additional optical and SEM images of SO-SLIPS and passivated soot; Figure S7: pictures of samples subjected to various damage tests; Figure S8: 3D height map of cuts damaged SO-SLIPS samples; Figure S9: sample force strain tests and optical profilometry; Figure S10: 3D FEM simulation of ice–3M tape system pulled along the longitudinal direction; Figure S11: 3D FEM simulation of ice–3M tape system pulled from the back of the ice column; Table S1: comparison of properties of several passive deicing surface technologies (PDF)
