# Supplementary material for: A Simple Approach for Flexible and Stretchable Anti-icing Lubricant-Infused Tape
Source: ACS Appl Mater Interfaces. 2021 Sep 8;13(37):45105–15. doi: 10.1021/acsami.1c15634 (PMC8461601; doi:10.1021/acsami.1c15634)
Supplement: Supplementary file 1 — am1c15634_si_001.pdf [file am1c15634_si_001.pdf]

Supporting Information

A simple approach for flexible and stretchable anti-icing lubricant-infused tape

Marco Carlotti, Ilaria Cesini and Virgilio Mattoli**

Dr. M. Carlotti, Dr. I. Cesini, Dr. V. Mattoli

Italian Institute of Technology, Center for Center for Materials Interfaces, Viale Rinaldo Piaggio 34, Pontedera, Italy

E-mail: marco.carlotti@iit.it; virgilio.mattoli@iit.it

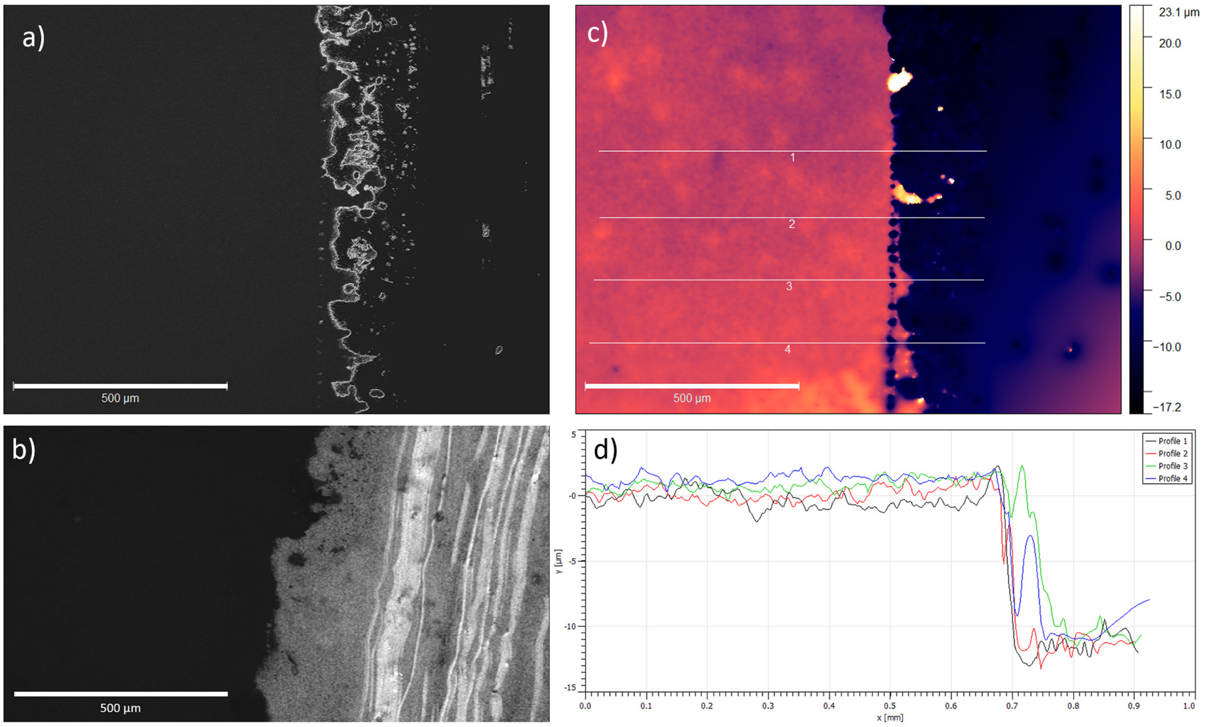

Figure S1. Optical profilometry analysis of candle soot deposited on double sided tape and scratched with a plastic rod: multifocal optical image (a), height map (b), bright field optical image (c), and height profile for the several lines indicated in panel c (d). Prior to the measurement, the soot sample was sputtered with a thin layer of gold to allow the focusing of the instrument on the dark soot region.

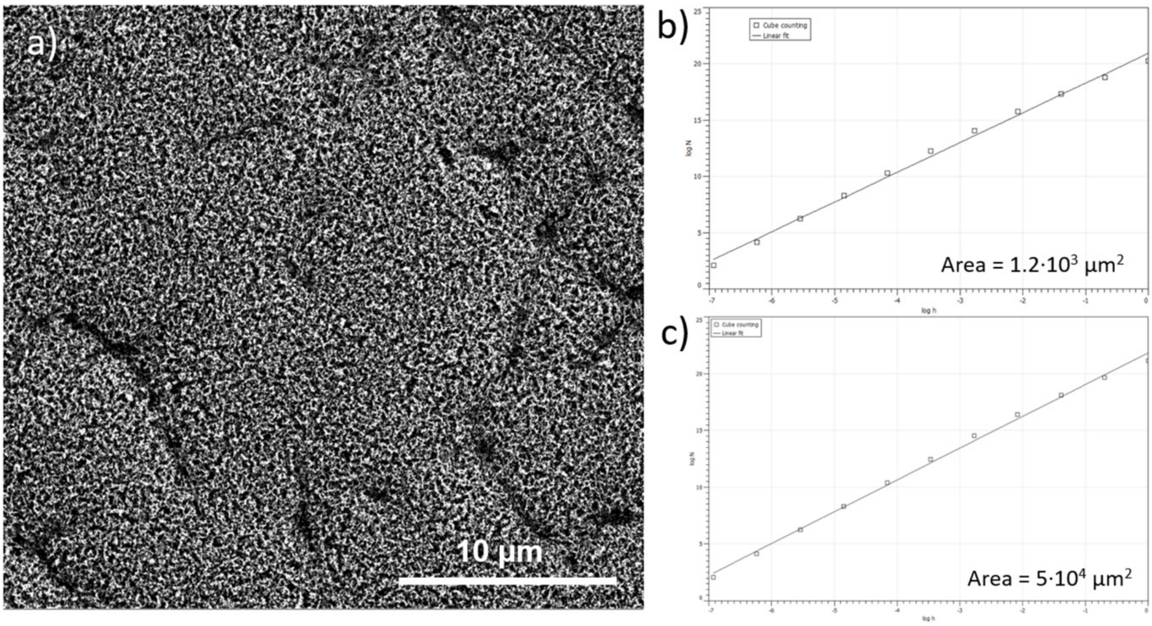

Figure S2. (a) SEM picture of deposited soot. (b, c) Cube counting method fitting for the determination of the fractal dimension.

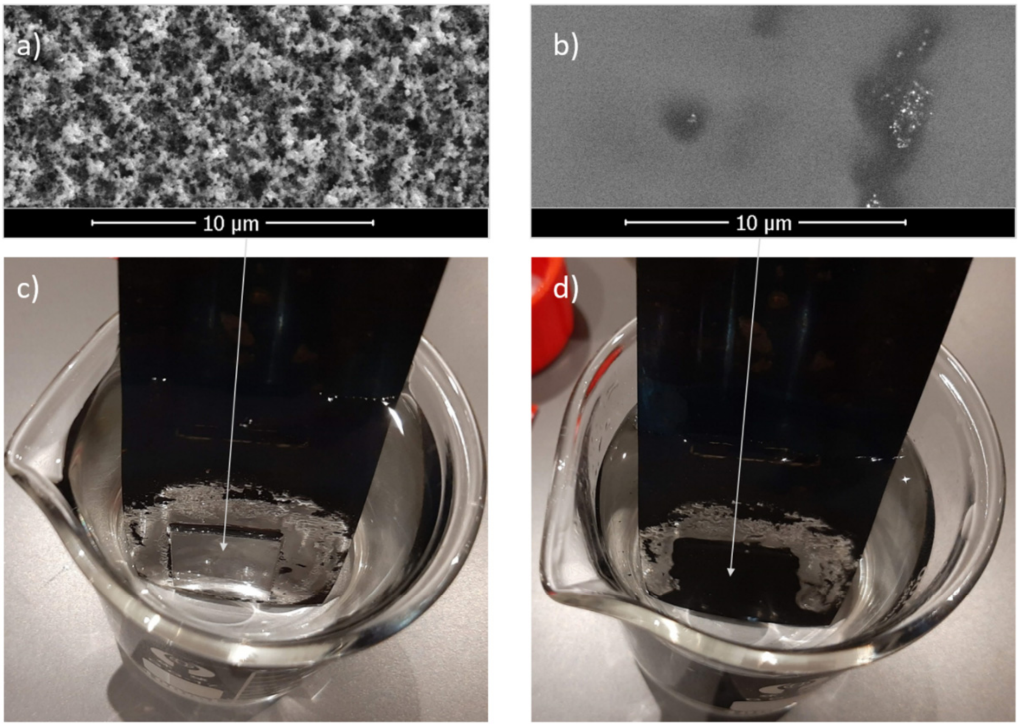

e)

Substrate	Contact Angle (°)
Soot	> 160
3M	107 ± 2
Silicone Oil	107 ± 0.5
Halocarbon	106 ± 3
Passivated + Silicone Oil	106 ± 1
Passivated + Halocarbon	85 ± 1

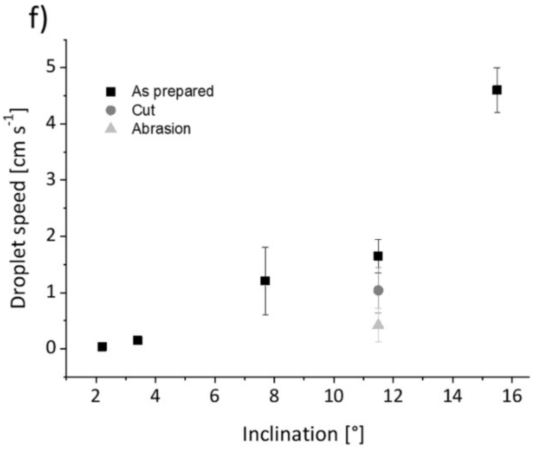

Figure S3. SEM images of deposited candle-soot (a) and SO-SLIPS surface (b). c) Example of Cassie-Baxter effect for soot-covered 3M tape. d) The 'mirror'-effect disappears in the case of SLIPS. e) Table summarizing the measured static water contact angles on the different substrates reported in Figure 2i in the main text. f) Plot of droplets sliding speeds at different angles and on different damaged surfaces.

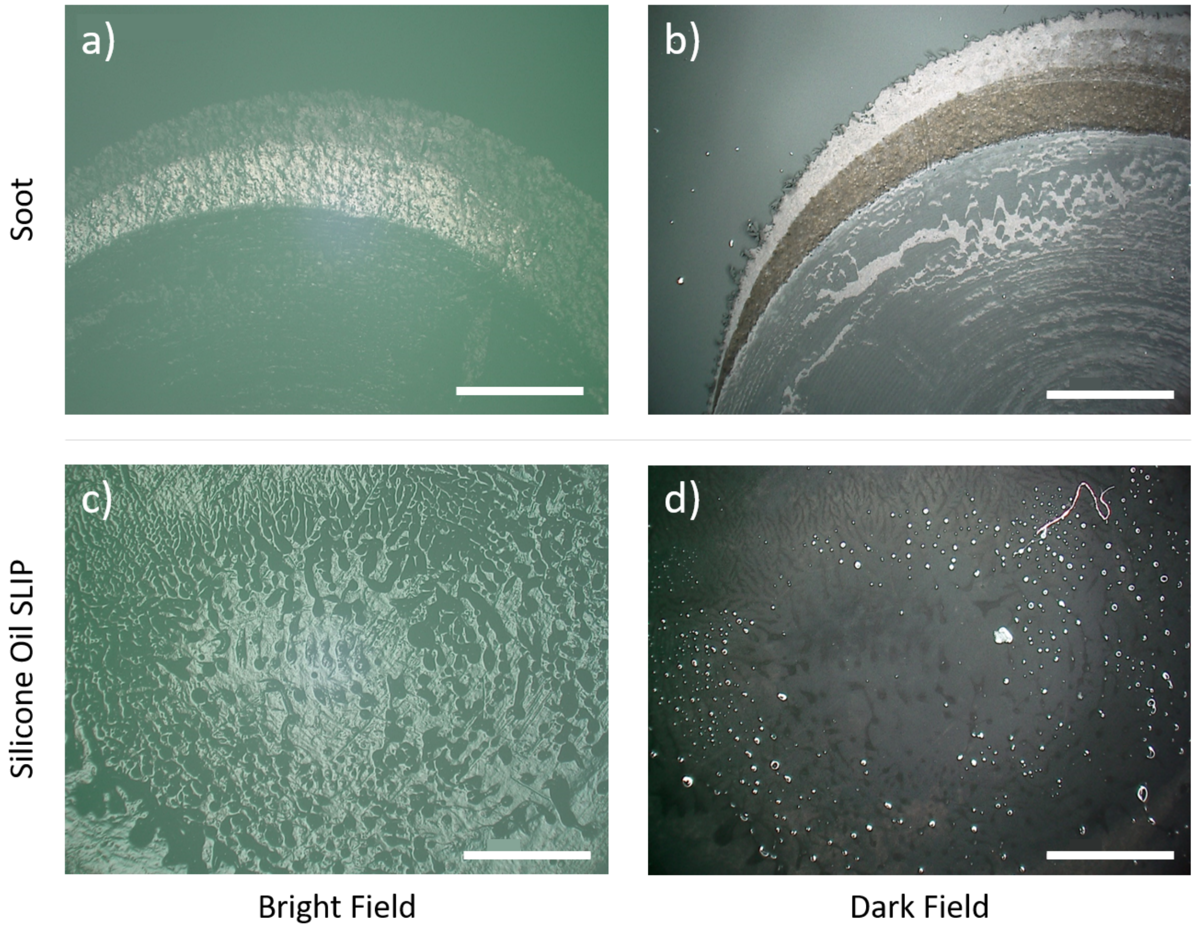

Figure S4. Optical microscopy images showing the effect of 100 kPa of pressure on deposited soot (a, b) and SO-SLIPS (c, d). Scale bar is 2 mm. While the pressure is sufficient to remove and deform the soot, the same does not happen when the lubricant is present. The defects seen on the surface are mostly due to the flowing of the lubricant to the surface due to the capillary forces between the metal rod used to apply the pressure and the surface.

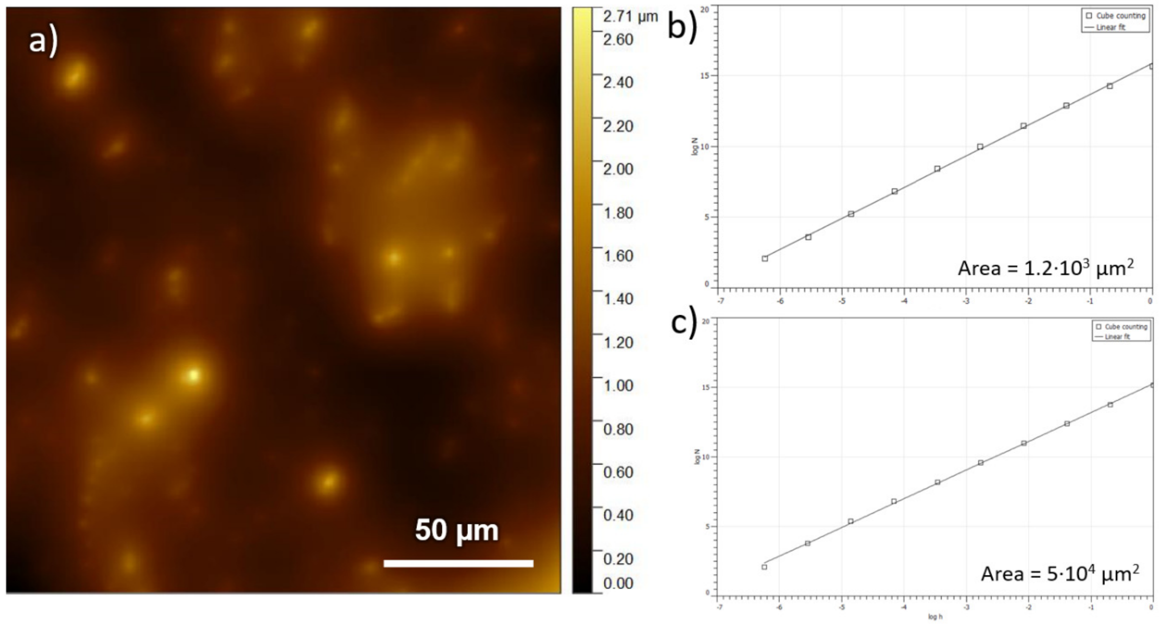

Figure S5. (a) Optical profilometer height map for SO-SLIPS. (b, c) Cube counting method fitting for the determination of the fractal dimension.

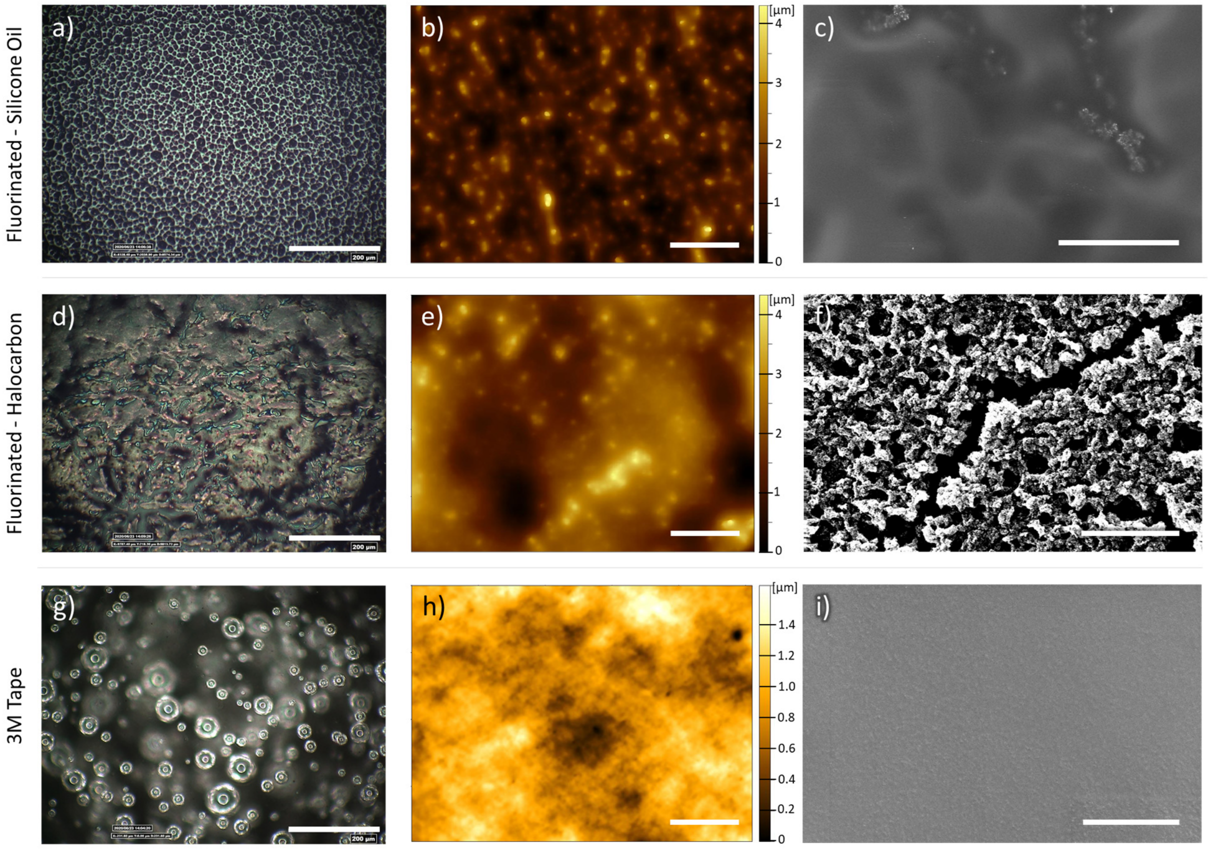

Figure S6. Images of SLIPS comprising silicone oil and passivated soot: optical microscopy (a), optical profilometer height map (b), and SEM image (c). Images of SLIPS comprising halocarbon and passivated soot: optical microscopy (d), optical profilometer height map (e), and SEM (f). Images of 3M tape: optical microscopy (g), optical profilometer height map (h), and SEM (i). Scale bars represent 200 µm, 50 µm, and 10 µm for optical microscopy images, profilomter height maps, and SEM pictures respectively.

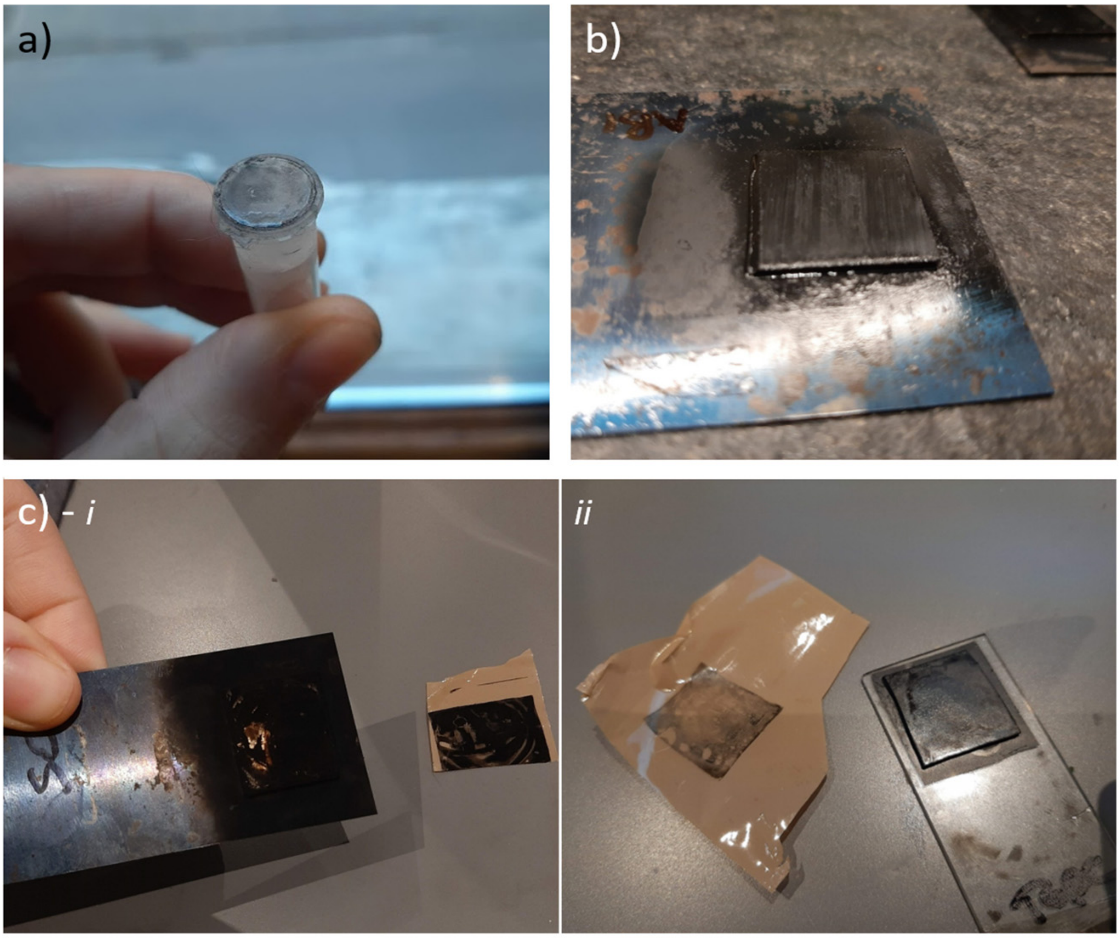

Figure S7. Photographs of: (a) residual soot after removal of the ice column; (b) SO-SLIPS after abrasion test; (c) removal of soot with adhesive tape from a metallic substrate (i) and SO-SLIPS (ii).

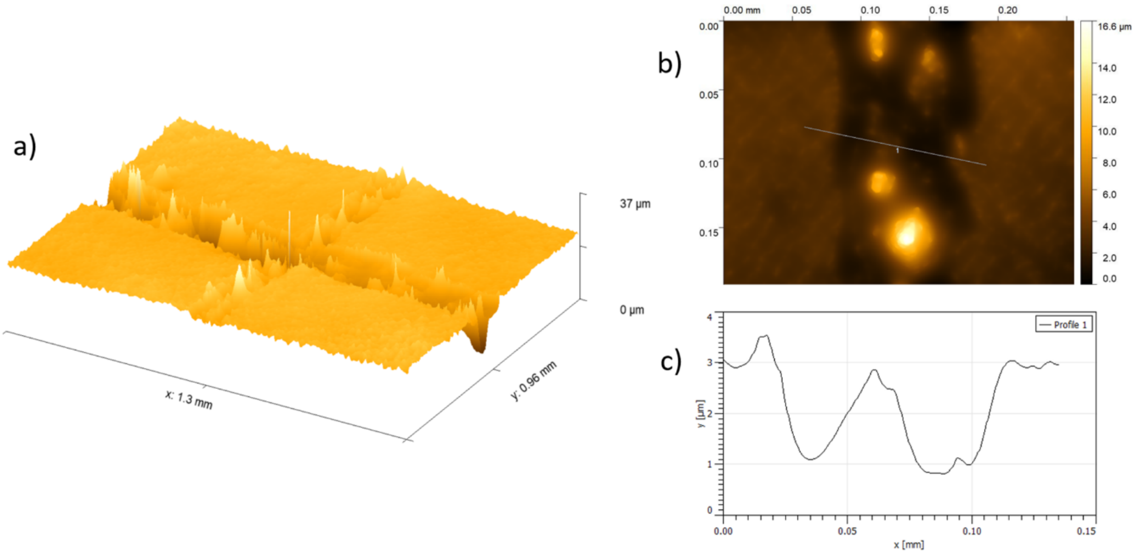

Figure S8. (a) 3D visualization of height map for two perpendicular cuts damage on SO-SLIPS. (b) 2D height map for a particular cut. (c) 1D depth profile for the white line in (b).

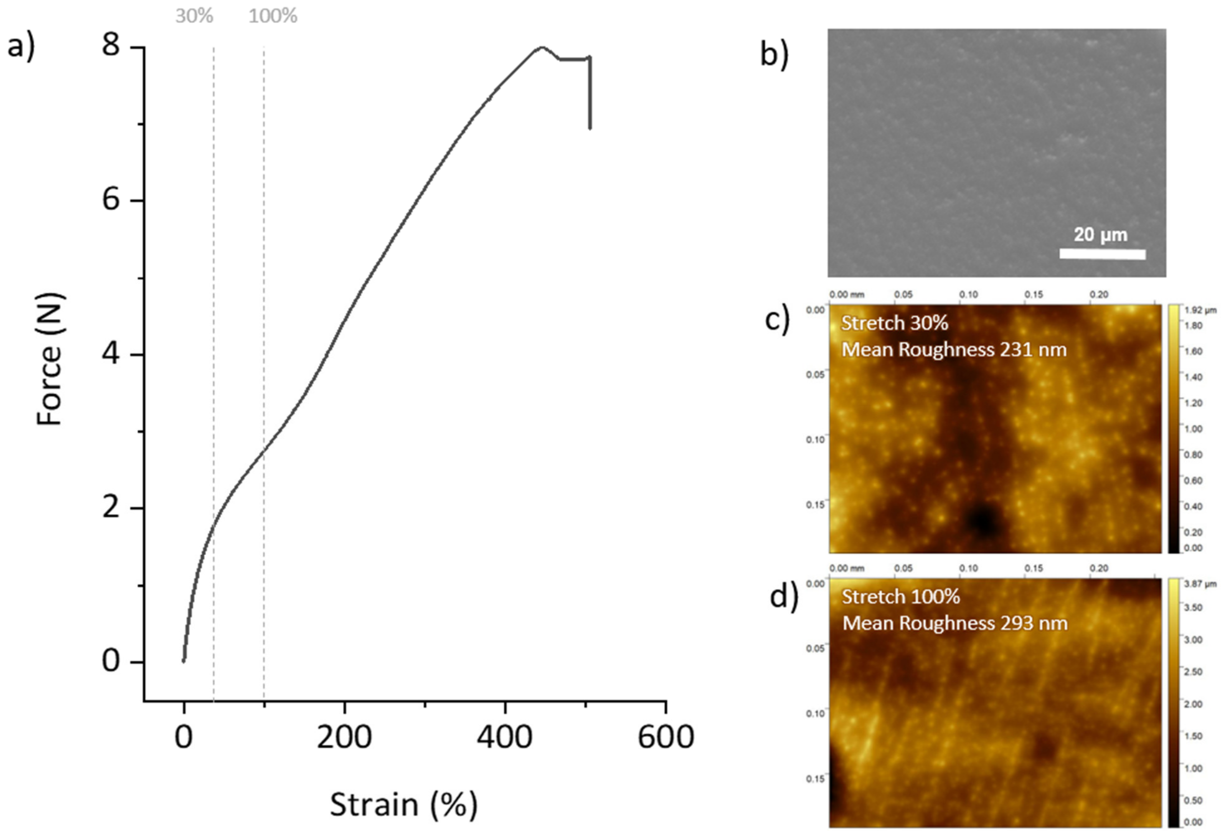

Figure S9. Force-strain plot for 3M tape (a). SEM image of SO-SLIPS after 3 stretching cycles to 30% (b). Optical profilometer height maps for SO-SLIPS after 3 stretching cycles to 30% (c) and 100% (d).

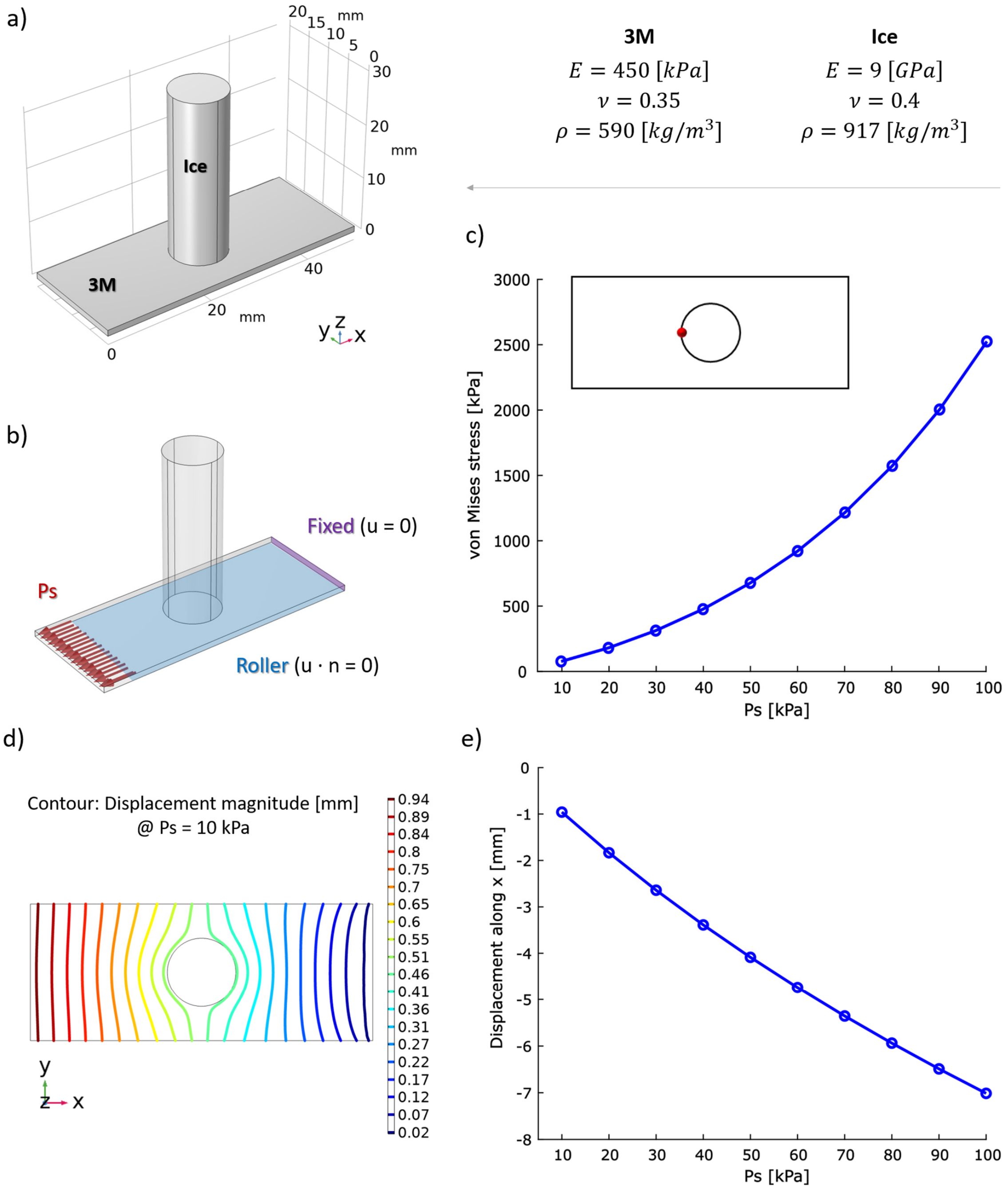

Figure S10. 3D Solid Mechanics model of the ice-3M tape system pulled at constant pressure along the longitudinal direction. (a) Geometry and material properties. (b) Boundary conditions. (c) Von Mises stress at the ice-tape interface as a function of the applied pressure varying in the 10-100 kPa range. (d) Contour plot of the longitudinal displacement magnitude of the tape when the tape is pulled at 10 kPa. (e) Evolution of the longitudinal displacement as a function of the applied pressure.

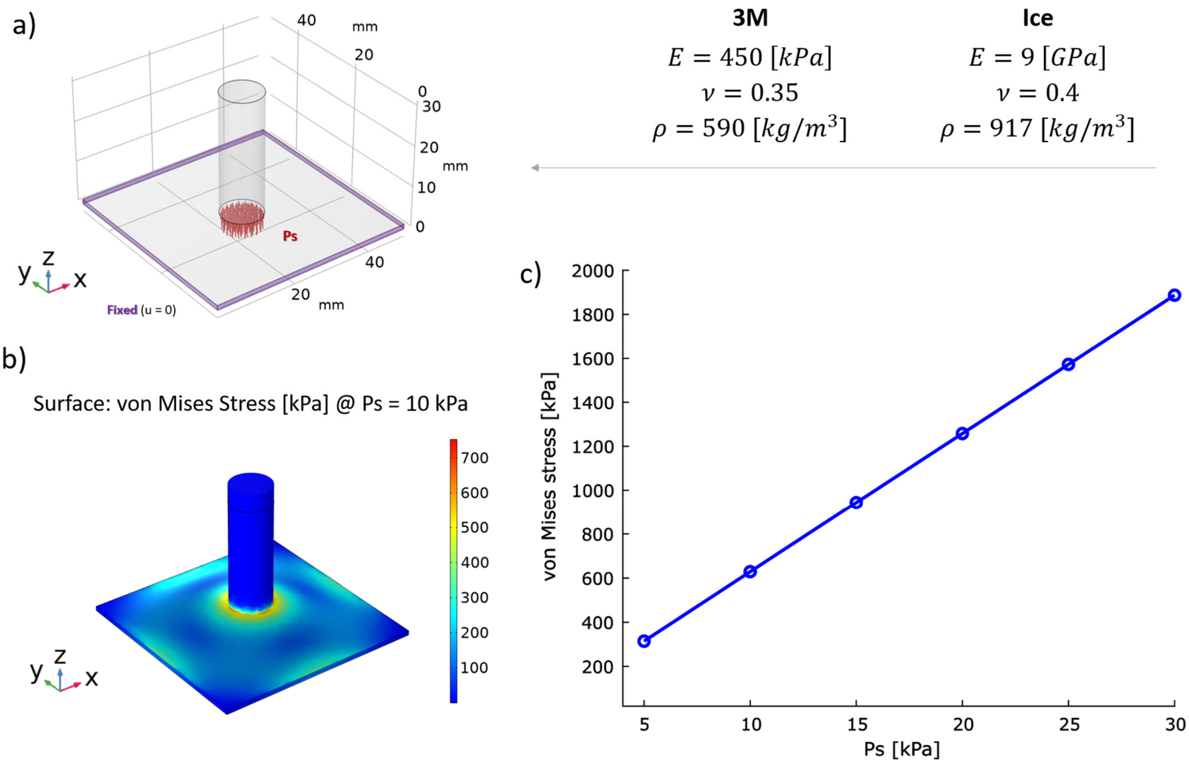

Figure S11. Finite element simulation of the ice-3M tape system pulled from the back of the ice column. (a) Geometry, material properties and boundary conditions of the system loaded with Ps varying in the 5-30 kPa range. (b) Von Mises stress distribution at 10 kPa. (c) Von Mises stress at the ice-tape interface as a function of the applied pressure.

Table S1. Comparison of properties of several passive de-icing surface technologies [numbering refers to the references in the main text].

	Working principle	Ice adhesion <20 kPa	Simple fabrication	Conformability	Stretchability	Resistance to damage
Coatings[18,21]	Inherent low ice adhesion	(X)	✓	✓	(✓)	✓
Self-assembled monolayers[14,15]		✓	X	✓	X	X
Superhydrophobic surfaces (SHSs) [16,17,19,20,46]	Lowering of freezing point	X	(✓)	(X)	X	✓
Anti-freeze containing gels[30,33]		(X)	X	✓	X	(✓)
Lubricant infused coatings[11]	Slippery surface	✓	X	✓	X	(X)
Alkane Infused Gels (AIGs) [25-27]		✓	(✓)	(✓)	(✓)	(✓)
Slippery Liquid-Infused Porous Surfaces (SLIPS) [28,38,39,47]		✓	(✓)	(✓)	X	(✓)
This Work		✓	✓	✓	✓	(✓)
